# Efficacy of a Transitional Support Program Among Adolescent Patients With Childhood-Onset Chronic Diseases: A Randomized Controlled Trial

**DOI:** 10.3389/fped.2022.829602

**Published:** 2022-03-31

**Authors:** Mayumi Morisaki-Nakamura, Seigo Suzuki, Asuka Kobayashi, Sachiko Kita, Iori Sato, Miwa Iwasaki, Yoichiro Hirata, Atsushi Sato, Akira Oka, Kiyoko Kamibeppu

**Affiliations:** ^1^Department of Family Nursing, Division of Health Sciences and Nursing, Graduate School of Medicine, The University of Tokyo, Tokyo, Japan; ^2^Department of Health Quality and Outcome Research, Global Nursing Research Center, Graduate School of Medicine, The University of Tokyo, Tokyo, Japan; ^3^Department of Pediatric Nursing, Tokyo Medical University, Tokyo, Japan; ^4^Department of Health Policy, National Center for Child Health and Development, Tokyo, Japan; ^5^Department of Nursing, The University of Tokyo Hospital, Tokyo, Japan; ^6^Department of Pediatrics, Kitasato University School of Medicine, Sagamihara, Japan; ^7^Department of Pediatrics, Graduate School of Medicine, The University of Tokyo, Tokyo, Japan; ^8^Saitama Children’s Medical Center, Saitama, Japan

**Keywords:** transitional support, transition readiness, childhood-onset chronic diseases, adolescent patients, randomized controlled trial

## Abstract

It is recommended that patients with childhood-onset chronic diseases (CCD) be transferred from pediatric to adult healthcare systems when they reach adulthood. Transitional support helps adolescents with CCD transition smoothly. Transition readiness is one of the key concepts to assess the efficacy of transitional support programs. This study aims to investigate the effect of a transitional support program on transition readiness, self-esteem, and independent consciousness among Japanese adolescents with various CCD using a randomized controlled trial. Adolescents with CCD aged 12–18 years participated in a randomized controlled trial evaluating the efficacy of a transitional support program. The patients in the intervention group visited transitional support outpatient clinics twice. They answered questionnaires regarding their disease and future perspectives to healthcare professionals and independently made a short summary of their disease. All the participants answered the questionnaires four times. Eighty patients participated in this study. Among those in the intervention group, transition readiness within one, three, and 6 months after interventions, and self-esteem within 1 month after interventions were higher than that of the control group. The scores on the “dependence on parents” subscale at 6 months after interventions were lower for the intervention group as compared to the control group. This program is expected to help patients transition smoothly from pediatric to adult healthcare systems.

## Introduction

The number of patients with childhood-onset chronic diseases (CCD) such as congenital heart disease, childhood cancer, diabetes, and epilepsy has increased, along with advancements in medical and surgical care (1, 2). The mortality rate of patients with CCD aged 1–19 years was 10.46 (per 100,000 population) in 1975 and 3.12 (per 100,000 population) in 2008 (3). However, these patients often develop complications in adulthood due to age-related changes in the treatment region, decline in treatment adherence, and development of lifestyle-related diseases (4–6). Therefore, they need to continue visiting the hospital regularly from childhood to adulthood, and transfer from pediatric to adult healthcare systems based on their age and physical and mental development (7).

Transitional support has been the focus for facilitating a smooth transfer from pediatric to adult healthcare systems. Patients with CCD tend to stop regular hospital visits, based on their own judgment, during the transitional period. Previous studies indicated that 39–65% of patients with congenital heart disease stopped visiting the adult cardiology outpatient clinic in the transitional period (8–10). The reasons included confusion and concern associated with moving to a different healthcare system (11), insufficient explanation by healthcare professionals, and patients’ poor understanding of the importance of regular and long-term hospital visits (12). Patients cannot receive optimal medical care and appropriately-timed interventions if they stop regular hospital visits, which may lead to a worsened prognosis (12, 13). Providing transitional support for patients, assessing their readiness for transfer to adult healthcare systems, and judging whether they can adapt to change before the transition is necessary to prevent a worsened prognosis owing to transfer from pediatric to adult healthcare systems.

Healthcare professionals should provide transitional support to enhance patients’ “transition readiness.” Transition readiness is the concept used by healthcare professionals to assess patients’ readiness to transfer to adult healthcare systems and to judge whether they will adapt to the change. This includes an acceptance and understanding of their own disease, and active disease management (14). It is suggested that improvement in transition readiness leads to a better ability to adapt to adult healthcare systems (14–16). A framework for youth with type 1 diabetes during their emerging adulthood transition indicated that transitional events related to various changes that a patient experienced during development directly affected health, developmental, and behavioral outcomes. Furthermore, it showed that these outcomes predicted a successful transition (17). According to this framework, the transitional support program as a transitional event is expected to improve patients’ transition readiness, a behavioral outcome predicting a successful transition.

Several previous studies have reported that transitional support programs improved transition readiness and various psychosocial outcomes among patients with CCD. A study of adolescents with congenital heart diseases revealed that face-to-face and online education by nurses about the disease and communication with healthcare professionals improved transition readiness and disease knowledge (18, 19). Another study with inflammatory bowel disease and type 1 diabetes patients also showed the improvement of their transition readiness, self-esteem, and patient-led communication through education by healthcare professionals *via* the Internet (20). Thus, transitional support programs based on disease-specific education by healthcare professionals with detailed disease knowledge have been developed and verified for efficacy. However, in situations where the facilities providing transitional support do not receive compensation from the medical system (such as in Japan), these validated programs, which are customized for a specific disease are unlikely to be accepted because of the lack of personnel for providing transitional support (21, 22).

Self-esteem and independent consciousness are also important psychological outcomes among patients with CCD in the transitional period. High self-esteem was related to high disease management among patients with CCD (23). Additionally, getting more information about the disease increased patients’ adaptation to society and improved their self-esteem (24). Among the adolescents and young adults with CCD, independent consciousness was lower and dependence on parents was higher than that of healthy peers (25). Several studies have also suggested lower independence among patients with CCD regarding disease management, including taking medicine and visiting hospitals (26, 27). Thus, self-esteem and independent consciousness are important aspects in patients with CCD in the transitional period and are expected to improve according to the change in transition readiness.

Adolescents’ independence is influenced in different ways by cultures and environments. A comparative study of parent–child relationships showed that Japanese youth experienced higher maternal control than their United States counterparts, owing to which the Japanese youth tended to be limited in their independence (28). Therefore, we need to support adolescent patients to be independent in their disease management, including their ability to communicate with healthcare professionals and visit doctors. Considering these difficulties in the capacity to provide transitional support as well as in the characteristics of patients, we need a transitional support program that can meet the needs associated with various CCD, can be managed with limited manpower and costs, and focuses on patients’ independence to enable an effective and sustainable support system.

The aim of this study was to investigate the effect of a transitional support program on transition readiness, self-esteem, and independent consciousness among Japanese adolescents with various CCD using a randomized controlled trial (RCT). We expected that the intervention of this study will improve the patients’ self-management skills by encouraging them to seek information about their disease and imagining future perspectives.

## Materials and Methods

### Study Design

This study used a non-blinded randomized controlled trial design.

### Eligibility Criteria

We recruited adolescent patients with CCD from a single university hospital in an urban area of Tokyo from July 2017 to January 2018. The inclusion criteria were as follows: (1) 12–18 years old, (2) able to converse with the researchers and answer questionnaires in Japanese, (3) visiting the pediatric outpatient clinic at the study facility regularly (every 1 month to one year), (4) able to assent to participate, and their guardian(s) provide consent to participate. People were excluded if: (1) they were judged by their attending doctor to have difficulty participating in the study because of mental vulnerabilities independent of intellectual or developmental disabilities, or acute physical symptoms and (2) were not informed of their diagnosis.

### Recruiting and Allocation

The patients who met all the inclusion criteria and their guardian(s) were invited to participate in the study by their attending doctor during a regular appointment. The researchers then explained the study purpose and obtained written informed assent from patients and informed consent from their guardian(s). If the patients visited alone, the researchers obtained only their written assent and asked them to pass on the research explanation and consent document to their guardian(s). The consent document signed by their guardian was returned to the researchers by mail. After obtaining informed consent, the patients were randomly allocated into the intervention or control group through a cloud-based program that can be allocated by participants through the generation of random numbers by a computer. We adopted a permuted block method (block size 4) using age and sex as allocation factors.

### Procedure

Participants answered questionnaire surveys including the Japanese version of the TRANSITION-Q, Rosenberg Self-Esteem Scale, Independent Consciousness Scale, and demographic information at four different time periods: T1 (within 1 month after agreeing to participate in this study), T2 (intervention group: within 1 month after intervention [the second transitional support outpatient clinic]; control group: 4 months after T1), T3 (3 months after T2), and T4 (6 months after T2). While the time periods of T1, T3, and T4 were uniform between the two groups that of T2 was different between the two groups. We could not keep the timing of T2 uniform in the intervention group because it depended on the schedule of the second transitional support outpatient clinic of each patient; it was based on the schedule of their regular visits to their attending doctor. We defined the periods because we expected an average duration between T1 and the second transitional support outpatient clinic to be 3 months based on our experience. Patients who failed to return questionnaires or provide answers online were reminded *via* letters or e-mails after two- and four-week intervals. As part of usual care, all participants received a leaflet describing the overview and importance of transition between T1 and T2. The leaflet described the explanation, importance, and timing of transition, and information on who would support patients’ transition.

### Intervention

A transitional support team consisting of pediatricians, nurses, psychologists, and nursing researchers reviewed and planned the intervention program. Contrary to previous RCTs validating transitional support programs, which included teleconference, Skype, web-based, and text-delivered interventions for participants, this study limited interventions to face-to-face consultations with healthcare professionals (18–20). In addition to regular visits to their attending doctor (usual care), the patients in the intervention group participated in transitional support outpatient clinics twice that were 20–30 min in duration. The transitional support program in this study included three important aspects: (1) the patients attended a transitional support outpatient clinic without their guardian(s), (2) healthcare professionals asked the patients questions using a common inquiry sheet, (3) the patients were asked to make “my health passport” to summarize the information of their disease after the first visit. The two visits were tailored to coincide with the patients’ regular appointments to avoid additional absence from school. At both the clinic visits, pediatricians and nurses asked them questions relating to their basic information, diagnosis, treatments, daily life, and future perspectives, using a common inquiry sheet. We expected that in answering these questions, the patients would reflect on their understanding of the disease, its self-management, and imagine their future. If patients could not answer a question, we encouraged them to ask or consult with their attending doctor about the question before the next visit. In the first clinic visit, patients were asked to fill “my health passport” at home and bring it to the second clinic visit. My health passport consisted of three sections: basic patient information, information about the disease, and things they want people around them to help with. The basic patient information included the patient’s date of birth, address, hospital and its contact information, the name and department of their attending doctor, emergency contact information, and information on the social security system or disability certificate. Information about the disease included the patient’s diagnosis, treatment received in the past, treatment currently being received, precautions in daily life, and precautions to be taken in the future. In the last column, the patients were asked to describe how they would like the people around them to respond to their disease and treatment (e.g., what they would like them to do in the event of seizure). The above information was directly filled out by the patient on a sheet of paper. At the second transitional support outpatient clinic, we asked the patients how they had gathered the information to complete their “my health passport” and whether they had any difficulties completing it. We asked the patients to confirm with their attending doctor whether the information they completed was correct.

### Outcome Measures

The primary outcome of this study was transition readiness at T2 which indicated the immediate effect of the transitional support program.

#### Transition Readiness

Transition readiness was measured with the TRANSITION-Q-J, the Japanese version of the TRANSITION-Q (16, 29). TRANSITION-Q-J was validated and comprises two subscales: communication and self-management and examination behavior (29). The scale includes items such as, “I ask the doctor or nurse questions” and “I contact a doctor when I need to.” Responses are rated on a three-point Likert scale (2 = always to 0 = never). The total score is converted to a 0–100-point scale using the score chart (which is based on the Rasch theory) originally calculated by the author and developer of TRANSITION-Q. Higher scores indicate higher transition readiness. In this study, Cronbach’s α was 0.84 for the TRANSITION-Q-J scale, 0.81 for the communication and self-management subscale, and 0.79 for the examination behavior subscale.

#### Self-Esteem

We used the 10-item Rosenberg Self-Esteem Scale (RSES) to assess patients’ self-esteem. Self-esteem was defined as “the sense that evaluates and trusts oneself as worthy of existing” and measured using a validated Japanese version of RSES (RSES-J) (30, 31). Examples of items in this scale are “I feel that I am a person of worth” and “I wish I could have more respect for myself.” A four-point Likert scale (1 = strongly disagree to 4 = strongly agree) is used to measure responses; higher total scores indicated higher self-esteem. In this study, Cronbach’s α for the RSES-J was 0.76.

#### Independent Consciousness

The Independent Consciousness Scale is a validated measure and comprises three subscales: independence, dependence on parents, and resistance and confusion. It evaluates the transition from dependence to independence among adolescents and young adults (32). The independence subscale comprises 10 items (e.g., “I can take responsibility for my own judgment”) including five items that are reverse scored; dependent on parents (e.g., “I want to depend on my parents when I am in trouble”) and resistance and confusion (e.g., “I often feel inferior to adults”) comprise five items each. A five-point Likert scale (1 = strongly disagree to 5 = strongly agree) is used to measure responses. In this study, Cronbach’s α was 0.79, 0.78 and 0.64 for each subscale.

### Participant Demographics

We collected data on sex, age, disease, school, medical treatment at home, and the frequency of the patients’ regular visits to their attending doctor using questionnaires and medical record surveys.

### Sample Size Calculation

We calculated the sample size for a two-group comparison without considering the effect of covariates because data predicting the effect of covariates were unavailable. The mean TRANSITION-Q total score was 52.74 ± 12.40 in patients with childhood-onset chronic diseases aged 12–18 years, who did not receive a transitional support program (16). In addition, based on a randomized controlled trial of a transitional support program, we estimated that the score in the self-management domain of the Transition Readiness Assessment Questionnaire (TRAQ), a measurement to assess transition readiness that is different from the TRANSITION-Q, increased by approximately 0.7 SD after intervention (19). A total of 66 participants in both groups were calculated to increase the TRANSITION-Q-J score by 8.7 points (0.7 SD), with a power of 80 and an alpha of 0.05 (two-sided test), using R version 1.37 (R Foundation for Statistical Computing, Vienna, Austria). The dropout rate in previous studies ranged from 5.0 to 11.5% (19, 20); we predicted a higher dropout rate than previous studies because the design of this study required two visits to the transitional outpatient clinic to participate in the program. Finally, we set the sample size as 40 participants in each group, with a total of 80 participants.

### Statistical Analyses

R Version 1.37 (R Foundation for Statistical Computing, Vienna, Austria), IBM SPSS 25.0 J for Windows (SPSS, Chicago, IL, United States), and IBM SPSS Amos ver. 25.0 (SPSS, Chicago, IL, United States) were used for analyses, and *p* < 0.05 (two-tailed) was considered significant. We included data from patients who deviated from the study protocol but did not include the data from patients who dropped out. Descriptive statistics were calculated for the demographic and disease variables. We compared the intervention group and control group using Mann–Whitney’s *U* test and Fisher’s exact test. For each outcome, we compared the two groups at each time using analysis of covariance (ANCOVA), as the baseline was covariate. In the event of an interaction between the independent variable (group) and covariate (at baseline) by parallelism test, we compared the groups using analysis of variance (ANOVA).

### Ethical Considerations

This study was approved by the Ethics Review Committee of the University of Tokyo, School of Medicine and registered at the University Hospital Medical Information Network Clinical Trials Registry (UMIN000028997). Both the patients and their guardian(s) provided oral and written consent. All participants received a leaflet describing an overview of transition, along with elaborating on its importance, to reduce the potential disadvantages for the patients in the control group. In addition, researchers explained to the patients in the control group that they could withdraw their assent for this study and participation in the transitional support outpatient clinic at any time.

## Results

### Participants’ Characteristics

**FIGURE 1:**
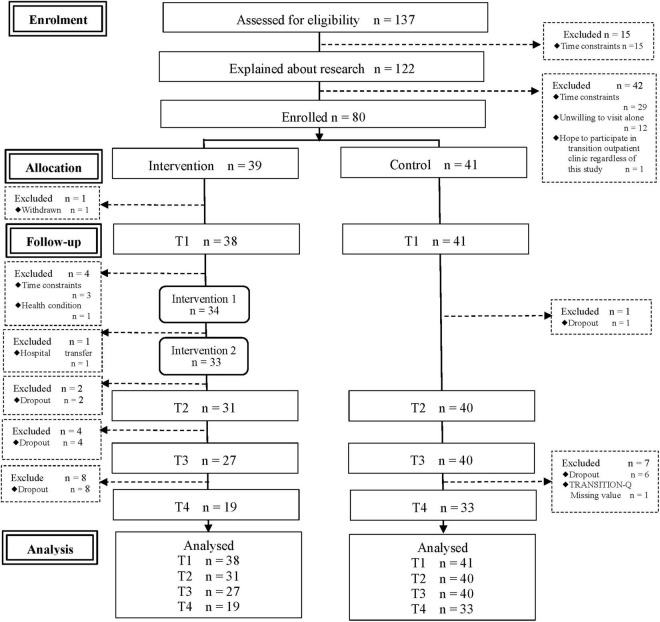
Flow diagram of the progress through the phases of a parallel randomized trial of two groups.

Of the 137 eligible candidates, 122 were provided details about this study from the researcher after their visit to their attending doctor. Eighty agreed to participate (participation rate 65.6%): 39 patients were allocated to the intervention group and 41 to the control group (Figure 1).

Demographic data are presented in Table 1. The participants’ mean age was 14.7 ± 1.8 years in the intervention group and 14.4 ± 2.1 years in the control group. The major disease categories were cardiology: 34 (43.0%); hematology and oncology: 15 (19.0%); neurology: 13 (16.5%); nephrology: 8 (10.1%); pediatric surgery: 4 (5.1%); allergy and immunology: 3 (3.8%); and endocrinology: 2 (2.5%). Two (2.5%) participants had a developmental disorder and two (2.5%) had an intellectual disability. Sixty-one (77.2%) participants were taking medication (Table 1). Despite randomization, all four patients with developmental disorders or intellectual disabilities were included in the intervention group. Furthermore, at T2, where primary outcomes were assessed, there were no significant differences in patient’ backgrounds between participants who continued to participate in the study and those who dropped out.

**TABLE 1:** Participants’ characteristics.

	Intervention (*n* = 38)		Control (*n* = 41)		
	*n* (%) or M ± SD	Range	*n* (%) or M ± SD	Range	*p*
Sex					
Male	25 (65.8)		25 (61.0)		0.816*^a^*
Female	13 (34.2)		16 (39.0)		
Age	14.7 ± 1.8	[12–18]	14.4 ± 2.1	[12–18]	0.465*^b^*
Disease					
Cardiology	16 (42.1)		18 (43.9)		0.685*^a^*
Hematology and oncology	7 (18.4)		8 (19.5)		
Neurology	7 (18.4)		6 (14.6)		
Nephrology	2 (5.3)		6 (14.6)		
Pediatric surgery	2 (5.3)		2 (4.9)		
Allergy and immunology	2 (5.3)		1 (2.4)		
Endocrinology	2 (5.3)		0 (0.0)		
Frequency of hospital visits					
Every 1–3 months	32 (84.2)		34 (82.9)		1.000*^a^*
Every 4 months or more	6 (15.8)		7 (17.1)		
Academic background					
Elementary school	3 (7.9)		3 (7.3)		0.862*^a^*
Junior high school	18 (47.4)		23 (56.1)		
High school	15 (39.5)		12 (29.2)		
Vocational school	2 (5.3)		2 (4.9)		
Other	0 (0.0)		1 (2.4)		
Intellectual disability	2 (5.3)		0 (0.0)		0.228*^a^*
Developmental disorder	2 (5.3)		0 (0.0)		0.228*^a^*
Home medical treatment					
None	7 (18.4)		9 (22.0)		0.783*^a^*
Medication	29 (76.3)		32 (78.0)		1.000*^a^*
Blood glucose self-monitoring or self-injection	2 (5.3)		3 (7.3)		1.000*^a^*
Exercise restriction					
None	22 (57.9)		21 (51.2)		0.340*^a^*
Light exercise	6 (15.8)		9 (22.0)		
Moderate exercise	6 (15.8)		3 (7.3)		
Prohibited from exercising	3 (7.9)		5 (12.2)		
Restriction on how they go to school	1 (2.6)		0 (0.0)		
Unknown	0 (0.0)		3 (7.3)		

*M, mean; SD, standard deviation;*

*^a^Fisher’s exact test;*

*^b^Mann–Whitney’s U test.*

The interval between T1 and T2 was 4.8 months for the intervention group and 4.2 months for the control group. There was no significant correlation between the interval from T1 to T2 and the difference in scores on TRANSITION-Q-J at T1 and T2 (ρ = 0.150, ρ = 0.216, respectively).

Of the 31 participants who attended the second transitional support outpatient clinic, 6 did not bring their “my health passports” with them and consequently filled them in during the session.

### Transition Readiness

**FIGURE 2:**
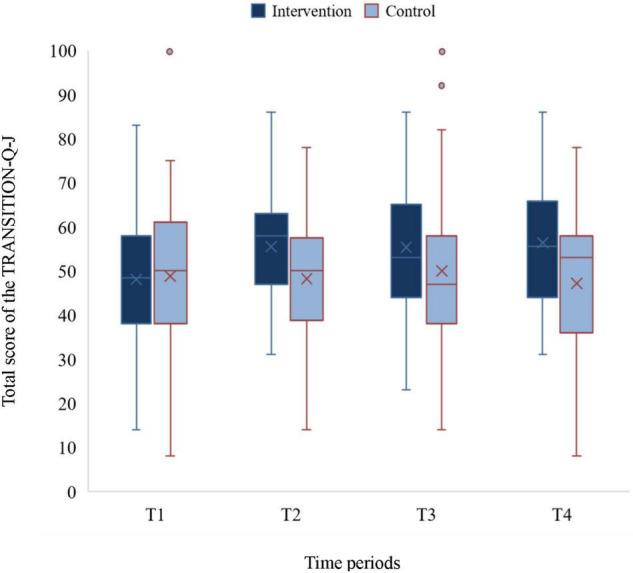
A box plot of the score trends.

There were no significant differences between the two groups in the total and subscale scores of TRANSITION-Q-J at T1. The total score of the intervention group showed an increase at T2, which was maintained until T4. In contrast, the score of the control group remained constant at all-time points. The total score of the intervention group was significantly higher than that of the control group at T2 (*F* = 8.45, *p* = 0.005; η^2^*_*p*_* = 0.11), T3 (*F* = 4.08, *p* = 0.048, η^2^*_*p*_* = 0.06), and T4 (*F* = 4.90, *p* = 0.032, η^2^_*p*_ = 0.09; Table 2, Figure 2). This was also true for the communication and self-management subscale score at T2 (*F* = 9.07, *p* = 0.004, η^2^*_*p*_* = 0.12), T3 (*F* = 4.77, *p* = 0.033, η^2^*_*p*_* = 0.07), and T4 (*F* = 4.17, *p* = 0.049, η^2^*_*p*_* = 0.08). However, no significant differences were found in examination behavior between the two groups at T2, T3, and T4.

**TABLE 2:** Differences in Transition Readiness as measured by the TRANSITION-Q-J.

	T1 (Intervention 38 vs. Control 41)				T2 (Intervention 31 vs. Control 40)					T3 (Intervention 27 vs. Control 40)					T4 (Intervention 19 vs. Control 33)				
	M	SD	*d*	*p*	M	SD	η *^2^_*p*_*	*F*	*p*	M	SD	η *^2^_*p*_*	*F*	*p*	M	SD	η *^2^_*p*_*	*F*	*p*
**TRANSITION-Q**																			
Intervention	48.05	15.50	0.04	0.859*^a^*	55.52	14.33	0.11	8.45	**0.005*^b^***	55.30	15.17	0.06	4.08	**0.048*^b^***	55.21	14.51	0.09	4.90	**0.032*^b^***
Control	48.78	16.93			48.20	15.10				50.02	18.15				47.15	16.05			
**Communication and self-management**																			
Intervention	10.92	3.54	0.11	0.640*^a^*	12.74	3.26	0.12	9.07	**0.004*^b^***	12.74	4.93	0.07	4.77	**0.033*^b^***	12.05	3.37	0.08	4.17	**0.049*^b^***
Control	11.34	4.13			10.80	3.86				11.17	4.06				10.48	4.20			
**Examination behavior**																			
Intervention	1.55	2.37	0.15	0.238*^a^*	2.45	2.78	0.02	1.24	0.270*^c^*	2.59	2.96	0.03	2.23	0.140*^b^*	3.05	3.22	0.03	1.28	0.264*^b^*
Control	1.90	2.40			1.90	1.91				2.10	2.78				2.24	2.61			

*M, mean; SD, standard deviation; d, Cohen’s d;η^2^_p_, partial η^2^;^a^Mann–Whitney’s U test;*

*^b^Analysis of covariance (ANCOVA);*

*^c^Analysis of variance (ANOVA). ANOVA was adopted for Examination behavior at T2 because the interaction between the independent variable (group) and the covariate (score at baseline) was significant by parallelism test. Bolded values are statistically significant (p < 0.05).*

### Self-Esteem

No significant differences in self-esteem were found between the two groups at T1. However, self-esteem in the intervention group was significantly higher than that in the control group at T2 (*F* = 4.54, *p* = 0.037, η^2^*_*p*_* = 0.07; Table 3). There were no significant differences between the groups at T3 and T4.

**TABLE 3:** Differences in self-esteem between groups as measured by the Rosenberg Self-esteem Scale.

	T1 (Intervention 37 vs. Control 39)				T2 (Intervention 29 vs. Control 38)					T3 (Intervention 23 vs. Control 38)					T4 (Intervention 18 vs. Control 33)				
	M	SD	*d*	*p*	M	SD	η *^2^_*p*_*	*F*	*p*	M	SD	η *^2^_*p*_*	*F*	*p*	M	SD	η *^2^_*p*_*	*F*	*p*
Rosenberg Self-Esteem Scale																			
Intervention	24.97	4.89	0.24	0.291*^a^*	26.34	5.49	0.07	4.54	**0.037^b^**	26.22	6.22	<0.01	0.32	0.572*^b^*	26.33	4.89	0.03	1.54	0.221*^b^*
Control	26.18	5.14			25.37	5.36				26.37	4.58				25.48	5.67			

*M, mean; SD, standard deviation; d, Cohen’s d;η^2^_p_, partial η^2^;*

*^a^Mann–Whitney’s U test;*

*^b^Analysis of covariance (ANCOVA). Bolded values are statistically significant (p < 0.05).*

### Independent Consciousness

The two groups did not show any significant differences across all three subscales of independent consciousness at T1, T2 and T3. At T4 (*F* = 5.18, *p* = 0.027, η^2^*_*p*_* = 0.10), the score for the subscale of dependence on parents was lower in the intervention than the control group (Table 4).

**TABLE 4:** Differences in independence consciousness between groups as measured by the Independent Consciousness Scale.

	T1					T2						T3						T4					
	n	M	SD	*d*	*p*	n	M	SD	η *^2^_*p*_*	*F*	*p*	n	M	SD	η *^2^_*p*_*	*F*	*p*	n	M	SD	η *^2^_*p*_*	*F*	*p*
**Independence**																							
Intervention	37	33.11	6.70	0.05	0.818*^a^*	29	33.59	6.18	<0.01	0.04	0.852*^b^*	23	34.17	6.52	0.01	0.55	0.461*^b^*	18	34.33	5.91	0.02	1.04	0.313*^b^*
Control	40	33.48	7.34			38	33.63	7.52				39	32.74	7.33				32	32.13	7.54			
**Dependence on parents**																							
Intervention	38	17.58	3.96	0.09	0.779*^a^*	30	17.60	3.54	<0.01	0.12	0.732*^b^*	24	17.08	3.79	0.04	2.41	0.125*^b^*	19	16.05	4.84	0.10	5.18	**0.027*^b^***
Control	41	17.22	4.29			39	17.56	4.04				40	18.53	3.88				33	18.48	3.56			
**Resistance and confusion**																							
Intervention	38	13.50	3.63	0.05	0.802*^a^*	30	13.47	3.76	0.01	0.75	0.391*^b^*	24	13.33	4.16	0.01	0.39	0.533*^b^*	19	13.68	3.27	<0.01	0.18	0.673*^b^*
Control	40	13.68	4.13			38	14.37	4.21				39	14.13	3.84				33	13.88	4.70			

*M, Mean; SD, Standard deviation; d, Cohen’s d;η^2^_p_, partial η^2^;*

*^a^Mann–Whitney’s U test;*

*^b^Analysis of covariance (ANCOVA). Bolded values are statistically significant (p < 0.05).*

## Discussion

The current study shows that the two-session transitional support outpatient clinics significantly enhanced and maintained transition readiness. Regarding secondary outcomes, self-esteem at T2 was higher in the intervention group than in the control group, and the dependence on parents’ subscale at T4 showed lower scores for the intervention group than for the control group. Our findings support the efficacy of the transitional support program used in this study among adolescents with CCD.

The important aspects of the transitional support program of this study were as follows: (1) patients attended transitional support outpatient clinics without their guardian(s), (2) healthcare professionals asked the patients questions using a common inquiry sheet, and (3) patients made a “my health passport” to summarize the information of their disease. For most participants, this was the first visit to an outpatient clinic without guardians. A patient’s transition readiness is enhanced through their own experiences of disease management, which had been handled by their guardian(s) for a long time (33). The intervention in this study provided an opportunity for patients to attend transitional support outpatient clinics without their guardian(s). This opportunity may have evoked awareness of their personal independence and improved transition readiness.

In the common inquiry sheet, we included questions regarding understanding of one’s own disease; the status of self-management; and perspectives regarding job selection, marriage, and having a child. There was a positive relationship between having future perspectives and transition readiness (34). Moreover, the support, which focused on their future, addressed their concerns, and encouraged active participation in decision-making improved the transitional process (21, 35). In this study, when patients answered these questions, they reflected on their understanding of the disease, disease self-management, and imagined their future. It was considered that patients thought about their disease in relation to their future perspectives and understood the need for independence and self-management within this context.

Becoming aware of various problems at transitional support outpatient clinics may provoke or increase concern in patients. Indeed, adolescent patients with CCD tend to believe that they have limited job opportunities. They are also likely more anxious about marriage, having a child, and the risk of their children inheriting their disease (36, 37). Therefore, healthcare professionals need to offer follow-up care and consultations to facilitate information processing regarding these issues.

Through the process of filling “my health passport,” the patients organized their disease information and realized their insufficient knowledge of the disease. The desire to know more about their own disease arises during adolescence, and by seeking information, adolescents with CCD are better equipped to face the future of living with their condition (37, 38). Further, seeking and selecting information fosters better decision-making and problem-solving skills among individuals living with CCD (38). Making a “my health passport” may also be considered as an experience of facing questions about their disease, which affected the changes in their attitudes. This included actively asking questions from healthcare professionals and seeking more information. The series of changes in the participants’ attitude toward their disease may consequently improve their transition readiness.

Most of the existing transitional support programs have deemed disease-specific education to be an important intervention for successful transitions (18–20). Hence, the existing programs need to be specifically designed for each disease or set of diseases, such as congenital heart disease, diabetes, and childhood cancer. Furthermore, these programs require professionals with sufficient knowledge and experience of each disease. Contrary to existing programs, in this study, we proposed a common transitional support program for patients with various diseases and expected to observe changes in patients’ information-seeking and examination behavior using a common inquiry sheet and the “my health passport,” which were used by adolescent patients with CCD. We established evidence of the efficacy of a new transitional support methodology that does not rely on individual disease-specific education.

Conversely, there is also a need to consider the method of intervention. In the present study, the dropout rate in the control group was 20%, while that in the intervention group was 51%. Notably, dropouts in T2, immediately after the interventions, were higher in the intervention group. In previous studies that introduced online or email-based interventions, the dropout rate ranged from 3–20% (19, 39, 40). While the mandatory face-to-face intervention is a unique and advantageous feature of this program, it is also possible that the manner of intervention was inconvenient for the patients. For adolescents, who have many commitments such as tests and examinations, sporting fixtures, cultural events, family responsibilities, as well as social gatherings and club activities, it is a heavy burden to visit the outpatient clinic for transitional support at a fixed time. Therefore, it is necessary to consider the possibility of visiting the outpatient clinic during school vacations. Furthermore, we need to devise how to evaluate the long-term effects of the intervention, such as allowing patients to respond at their regular outpatient visits in order to reduce the dropout rate.

As previous studies have shown (23, 24), the transitional support program was expected to improve patients’ self-esteem by answering questions from doctors and nurses, gathering information regarding their disease, and visiting the clinic without their guardian. Although the self-esteem scores for the intervention group at T2 were significantly higher than that for the control group, the difference in the two mean scores of the intervention and control groups was only 0.97 points. Further, at T2, the score decreased by 0.81 points for the control group. The significant differences between the two groups at T2 may have been affected by this decrease in the score of the control group. Thus, it is difficult to attribute the improvement in self-esteem in the intervention group at T2 to the transitional support received.

At T4, dependence on parents in the intervention group was significantly lower than that of the control group. Parents tended to continue taking initiative regarding their children’s disease management, and the patients often had limited independence because of their parents’ intervention (41, 42). The experience of getting the information from healthcare professionals by themselves, and that of conveying their understanding of their disease and future perspectives in the absence of their guardians may have enhanced patients’ independence.

In many countries including Japan, transitional support from pediatric to adult healthcare systems has not been established as an independent medical domain, and it is difficult to secure related costs and human resources (21). Our study provides a methodology to help establish a sustainable support system.

### Limitations

Although the study has several merits, some limitations must also be discussed. First, the intervention group had several dropouts because of the participants’ busy schedule, and only the patients who were willing to transition were included in the final analysis; thus, the intervention effect may have been overestimated. In the future, it is necessary to examine the timing and method of interventions and devise ways to reduce attrition.

Second, we did not assess the age of onset of the patients’ conditions and the duration of the chronic disease or compare it between the two groups even though it could have affected the patients’ understanding of their disease and their ability to cope with it. The time elapsed since the chronic disease diagnosis could have had an impact on the efficacy of the intervention.

Third, in this study, we could not uniform the interval from T1 and T2 between the two groups. Though we confirmed no significant correlation between the interval from T1 and T2 and the difference in scores on TRANSITION-Q-J at T1 and T2, the length of the response interval could affect the transition readiness scores since transition readiness is related to patients’ age (14–16). Therefore, future studies should uniform the interval of questionnaire surveys between the intervention and control groups.

Fourth, the result of self-esteem needs careful consideration. Although there was a significant difference in self-esteem between the two groups at T2, this difference was minute, and it may have depended on the decrease of the scores in the control group. In future studies, there is a need to investigate other factors related to possible changes in self-esteem.

Finally, as TRANSITION-Q is a scale that evaluates only the skills necessary to maintain health by subjective assessment. We should interpret the results of this study based on the understanding that it was limited to disease management and did not cover the general skills require for adolescents to be independent. Additionally, the efficacy of transitional support program could have been assessed using objective outcomes.

### Conclusion

We conducted a randomized controlled trial to reveal that the transitional support program in an outpatient setting is effective in enhancing transition readiness for CCD. This transitional support program that focused on patients’ independence is useful for patients with any disease, as well as for healthcare professionals who do not have specific and sufficient knowledge and experience regarding each disease. Further, this program would be feasible under settings with limited manpower and resources, including such settings in Japan.

## Data Availability Statement

The datasets analyzed in this article are not publicly available because participants of this study did not agree for individual data to be shared publicly. Requests to access the datasets should be sent to the corresponding author.

## Ethics Statement

The studies involving human participants were reviewed and approved by Ethics Review Committee of the University of Tokyo, School of Medicine. Written informed consent to participate in this study was provided by the participants and their legal guardian.

## Author Contributions

MM-N contributed to conception, design, intervention, data collection, statistics, and data analysis, and drafted the manuscript. MI, YH, and AS participated in designing the study, intervention, and data collection. SS and AK participated in designing the study, intervention, data collection, and statistical analysis. SK and IS participated in designing the study and statistical analysis. KK and AO supervised the whole study process, from the design of the study and intervention implementation to the completion of the manuscript. All authors participated in the critical revision and read and approved the final manuscript.

## Conflict of Interest

The authors declare that the research was conducted in the absence of any commercial or financial relationships that could be construed as a potential conflict of interest.

## Publisher’s Note

All claims expressed in this article are solely those of the authors and do not necessarily represent those of their affiliated organizations, or those of the publisher, the editors and the reviewers. Any product that may be evaluated in this article, or claim that may be made by its manufacturer, is not guaranteed or endorsed by the publisher.
